# Chondrogenic ATDC5 cells: An optimised model for rapid and physiological matrix mineralisation

**DOI:** 10.3892/ijmm.2012.1114

**Published:** 2012-08-31

**Authors:** P.T. NEWTON, K.A. STAINES, L. SPEVAK, A.L. BOSKEY, C.C. TEIXEIRA, V.E. MACRAE, A.E. CANFIELD, C. FARQUHARSON

**Affiliations:** 1 The Roslin Institute and R(D)SVS, University of Edinburgh, Easter Bush, Midlothian EH25 9RG;; 2 Wellcome Trust Centre for Cell-Matrix Research and Cardiovascular Research Group, Manchester M13 9PT, UK;; 3 Musculoskeletal Integrity Program, Hospital for Special Surgery, New York, NY;; 4 Basic Science and Craniofacial Biology, New York University, College of Dentistry, New York, NY, USA

**Keywords:** ATDC5, chondrocyte, mineralisation, β-glycerophosphate, endochondral ossification, growth plate

## Abstract

The development of chondrogenic cell lines has led to major advances in the understanding of how chondrocyte differentiation is regulated, and has uncovered many signalling pathways and gene regulatory mechanisms required to maintain normal function. ATDC5 cells are a well established *in vitro* model of endochondral ossification; however, current methods are limited for mineralisation studies. In this study we demonstrate that culturing cells in the presence of ascorbic acid and 10 mM β-glycerophosphate (βGP) significantly increases the rate of extracellular matrix (ECM) synthesis and reduces the time required for mineral deposition to occur to 15 days of culture. Furthermore, the specific expression patterns of *Col2a1* and *Col10a1* are indicative of ATDC5 chondrogenic differentiation. Fourier transform-infrared spectroscopy analysis and transmission electron microscopy (TEM) showed that the mineral formed by ATDC5 cultures is similar to physiological hydroxyapatite. Additionally, we demonstrated that in cultures with βGP, the presence of alkaline phosphatase (ALP) is required for this mineralisation to occur, further indicating that chondrogenic differentiation is required for ECM mineralisation. Together, these results demonstrate that when cultured in the presence of ascorbic acid and 10 mM βGP, ATDC5 cells undergo chondrogenic differentiation and produce a physiological mineralised ECM from Day 15 of culture onwards. The rapid and novel method for ATDC5 culture described in this study is a major improvement compared with currently published methods and this will prove vital in the pursuit of underpinning the molecular mechanisms responsible for poor linear bone growth observed in a number of chronic diseases such as cystic fibrosis, chronic kidney disease, rheumatological conditions and inflammatory bowel disease.

## Introduction

The growth plate and its primary cell type, the chondrocyte, are integral to endochondral ossification and thus the linear growth of the long bones (1). The continuing development of *in vitro* chondrocyte cell lines has furthered our understanding of the underlying mechanisms of endochondral ossification.

The ATDC5 cell line, which was first isolated from the differentiating teratocarcinoma stem cell line AT805, is commonly used as a model for *in vitro* chondrocyte research (2). To date, the ATDC5 cell line has been utilised in approximately 300 studies. Previous studies have detailed a well-characterised method of ATDC5 differentiation and mineralisation, initially by Shukunami *et al* (3). This method has provided a reliable model of *in vitro* chondrocyte mineralisation for a number of years and has been widely used in the field since its publication; however it does contain some drawbacks. For example, mineralisation studies require a culture time of at least 34 days and a change of culture conditions. Both the cell culture medium and the CO_2_ concentration have to be altered after 21 days of culture to facilitate extracellular matrix (ECM) mineralisation 13 days later. Since its publication, a number of groups have attempted to simplify the culture method. For example, the addition of inorganic phosphate to ATDC5 cultures has been shown to increase differentiation and the rate of ECM mineralisation (4,5). Another study has detailed that the addition of ascorbic acid shortened the proliferation phase of the ATDC5 cells from 21 to 7 days (6); while the temporal expression of markers of chondrogenic differentiation was examined, the ECM mineralisation capability of the ATDC5 cells under these culture conditions was not.

Therefore, our aim was to develop a culture model for ATDC5 cells which produced both consistent chondrogenesis and physiological ECM mineralisation in a reduced time period for *in vitro* experimentation. In this study, β-glycerophosphate (βGP) was added throughout the culture period. βGP is cleaved by alkaline phosphatase (ALP) and other phosphohydrolases produced by the chondrocytes once they have reached hypertrophy to release inorganic phosphate, thus mimicking the phosphate availability *in vivo* (3,7,8). It was hypothesised that this strategy would facilitate an incremental increase in mineral deposition once an appropriate ECM had been deposited. This would thereby increase the rate of mineralisation compared with previous methods while retaining the expected stages of chondrogenic differentiation as well as crucially, the formation of physiological mineral.

## Materials and methods

### Cell culture

Chondrogenic ATDC5 cells (Riken Cell Bank, Ibaraki, Japan) (3) were cultured in a differentiation medium [DMEM/F-12 (1:1) with GlutaMAX I containing 5% FBS, 1% insulin transferrin and selenium, 1% sodium pyruvate and 0.5% gentamicin (Invitrogen, Paisley, UK)] at a density of 6,000 cells/cm^2^ in multi-well plates (Iwaki Cell Biology; Sterilin, Feltham, UK) (9,10). Cells were left for 6 days to reach confluency at which point the medium was supplemented with 10 mM βGP and 50 μg/ml L-ascorbate-2-phosphate (ascorbic acid). Cells were incubated in a humidified atmosphere (37°C, 5% CO_2_) for up to 41 days and the medium was changed every second or third day. For levamisole experiments, ATDC5 cells were cultured in varying concentrations of levamisole (Sigma, Gillingham, UK) (0–1,000 μM) for up to 15 days.

### Histochemical staining

Calcium deposition in ATDC5 cells was evaluated by Alizarin red staining as described previously (11). Briefly, cells were fixed in 4% paraformaldehyde and then 2% Alizarin red (Sigma, pH 4.2) was added to the cell layers for 5 min at room temperature. Cells were washed with distilled water (dH_2_0) and images were captured. Alizarin red-stained cultures were extracted with 10% cetylpyridinium chloride for 10 min and the optical density (OD) of the digests was measured at 570 nm by spectrophotometry (Multiskan Ascent; Thermo Electron Corporation, Vantaa, Finland). Proteoglycan synthesis was evaluated by staining the cell layers with Alcian blue (Sigma). Cells were fixed in 95% methanol for 20 min and stained with 1% Alcian blue 8GX in 0.1 M HCl overnight. Cells were washed in dH_2_0 and images were captured. Alcian blue-stained cultures were extracted with 6 M guanidine-HCl for 6 h at room temperature and the OD was determined at 630 nm by spectrophotometry (11).

### Real-time quantitative PCR (qRT-PCR)

RNA was extracted using the RNeasy Mini kit (Qiagen Ltd., Crawley, West Sussex, UK), according to the manufacturer’s instructions. For each sample, total RNA content was assessed by absorbance at 260 nm and purity by A260/A280 ratios, and then reverse-transcribed. cDNA was diluted to 10 ng/μl in nuclease-free water (Sigma), and stored at −20°C. qRT-PCR reactions were conducted with a MX3000P qPCR machine (Stratagene, Stockport, UK) using a SYBR-Green detection method. Primers were designed in-house and synthesised by MWG Eurofins, London, UK. Reactions were run in triplicate and routinely normalised against GAPDH. Primer sequences: *Col2a1*, forward, 5′-CGGTCCTACGGTGTCAGG-3′ and reverse, 5′-GCAGAGGACATTCCCAGTGT-3′; *Col10a1*, for wa rd, 5′-CATA A AG G GCCCACT TGCTA-3′ and reverse,5′-CAGGAATGCCTTGTTCTCCT-3′; GAPDH forward, 5′-TGAGGCCGGTGCTGAGTATGTCG-3′ and reverse, 5′-CCACAGTCTTCTGGGTGGCAGTG-3′. qRT-PCR products were sequenced by the GenePool, University of Edinburgh.

### Transmission electron microscopy (TEM)

ATDC5 cells were cultured at 6,000 cells/cm^2^ on nitrocellulose discs (Nunc, Roskilde, Denmark) in mineralising conditions for 15 days. Cells were fixed in 2.5% glutaraldehyde in 0.1 M sodium cacodylate buffer at 37°C for 1 h. During processing, the cell monolayers were washed in 0.1 M sodium cacodylate, post-fixed in 1% osmium tetroxide and dehydrated through graded alcohols (35, 70, 95 and 100%). The monolayers were then processed to Epon in a vacuum oven at 60°C. Monolayers were viewed using a Phillips CMIRO TEM (FEI Vic Ltd., Cambridge, UK) and images were captured on Gatan Orius ICD camera (Gatan, Oxford, UK).

### Fourier transform-infrared spectroscopy (FTIR)

ATDC5 cells were cultured for 41 days in mineralising conditions as previously described. Cell monolayers were fixed in 95% methanol and embedded in LR White. Spectral images of 2 μm-thick culture sections were collected using a Spectrum Spotlight 100 system (Perkin-Elmer, Waltham, MA, USA) with a spectral resolution of 4 μm and 6.25 μm pixel size in transmission mode. The collected spectra were truncated, base-lined and the contribution of LR White was spectrally subtracted using ISYS software (Spectral Dimensions, Olney, MD, USA) and then analysed using ISys Chemical Imaging software. Spectra extracted from these images were analysed using Grams/32 software (Thermo Electron Corporation, Waltham, MA, USA). The parameters measured included mineral/matrix ratio, carbonate/phosphate ratio, crystallinity and collagen maturity (12).

### Statistics

Data were analysed by one-way analysis of variance (ANOVA), with Tukey simultaneous tests used to identify differences between individual time-points, using SigmaPlot 11.0 software (Systat Software UK Ltd., London, UK). Cell culture experiments were repeated at least twice and P<0.05 was considered statistically significant.

## Results

### ATDC5 cells undergo the expected stages of chondrocyte differentiation

**Figure 1.**
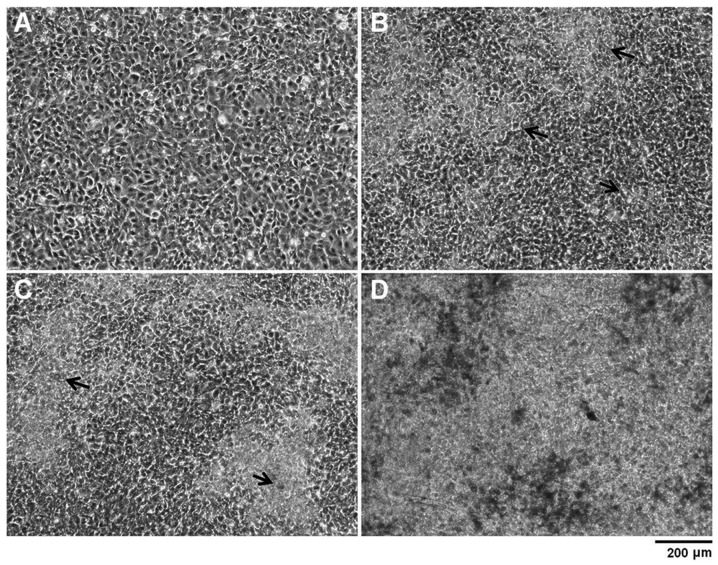
Chondrogenic differentiation of ATDC5 cells. ATDC5 cells were grown over a period of 34 days and light microscopy images were collected during this time period. (A) Image captured at confluence, Day 6 of culture. (B) Arrows indicate translucent ECM which begins to form in discrete locations at Day 13 of culture. (C) The arrows denote opaque regions which represent the onset of mineralisation within the ECM at Day 14. (D) The nodules of ECM conjoin over the ECM and mineralisation increases.

**Figure 2.**
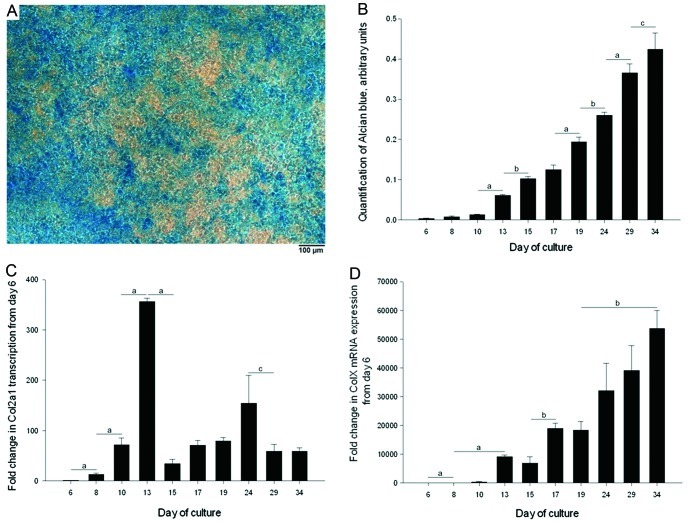
Chondrocyte marker analysis during ATDC5 cell differentiation. (A) The monolayer was fixed following 34 days of culture and stained for GAG using Alcian blue. (B) Quantification of Alcian blue staining was conducted over 10 time-points. (C) qRT-PCR analysis of *Col2a1* and (D) qRT-PCR analysis of *Col10a1* transcription, normalised to GAPDH, over 10 time-points in the ATDC5 monolayers. Mean (n=3) ± SEM, with the exception of *Col10a1* at Day 10 where n=2; ^a^P<0.05; ^b^P<0.01; ^c^P<0.001, where all significant differences between consecutive time-points are shown.

Images collected by light microscopy over a 34-day time-course indicated comparable differentiation to previously characterised ATDC5 cultures (3,4). ATDC5 cell cultures reached confluency 6 days after seeding with no extensive ECM formation (Fig. 1A). At this point, ATDC5 cells were then cultured in the presence of 10 mM βGP and 50 μg/ml ascorbic acid. This facilitates cell differentiation and the secretion of an extensive ECM which assembles around the cells as visualised at Day 13 of culture by the phase contrast images (Fig. 1B) and by Alcian blue staining (data not shown). As differentiation continues, these nodules increased in area and began to conjoin (Fig. 1C). A large proportion of each ATDC5 monolayer was Alcian blue-positive by Day 34 of culture, indicating that ATDC5 cells produced a glycosaminoglycan (GAG)-rich ECM and underwent chondrogenesis (Fig. 2A). The temporal increase in GAG-deposition over a 34-day time-course was established by quantifying Alcian blue staining (Fig. 2B). GAG-deposition progressively increased from Day 6, such that there were significant increases in Alcian blue staining between various time-points within the culture period.

To examine the process of chondrogenesis and hypertrophic differentiation of the ATDC5 cells during monolayer culture, *Col2a1* and *Col10a1* gene transcription at specific time-points was analysed by qRT-PCR. *Col2a1* transcription increased significantly between each time-point from Day 6 to 13; transcription then decreased but even at Day 34, transcription was greater compared to Day 6 (by >58-fold, P<0.001) (Fig. 2C). *Col10a1* transcription also increased significantly over the first 4 time-points up to Day 13 over a 10,000-fold range, which by Day 34 progressed to a greater than 50,000-fold increase in transcription compared to that of Day 6 (Fig. 2D).

### ATDC5 cells mineralise their surrounding ECM, producing physiological mineral

**Figure 3.**
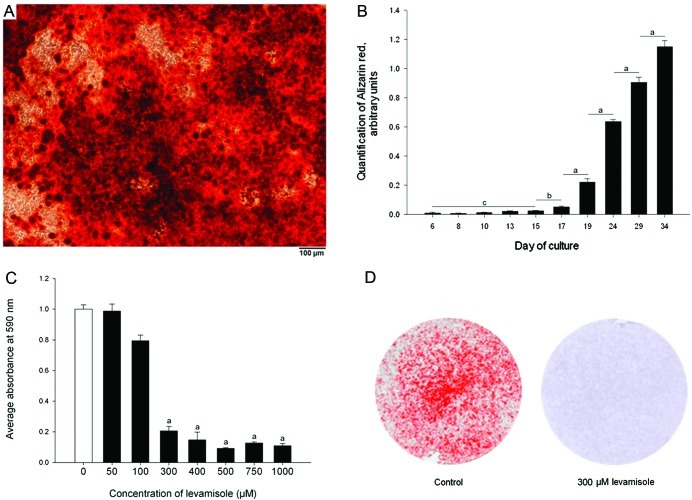
ATDC5 cell ECM mineralisation. (A) Mineralisation was examined by Alizarin red staining of monolayers fixed at Day 34 of culture. (B) Quantification of Alizarin red staining was conducted over 10 time-points. Mean (n=3) ± SEM; ^a^P<0.05; ^b^P<0.01; ^c^P<0.001, where all significant differences between consecutive time-points are shown. (C) Levamisole, an inhibitor of ALP enzyme activity, was added to ATDC5 cell cultures from when they reached confluency. Levamisole dose-dependently inhibited ATDC5 ECM mineralisation as indicated by quantification of Alizarin red staining. (D) Alizarin red stained images of the control cells and cells treated with 300 μM levamisole at Day 15 of culture. Data are represented as the means ± SEM (n=3 replicates) in comparison to 0 μM levamisole ^c^P<0.001.

Phase contrast images indicated mineralisation of the ATDC5 ECM from Day 14 onwards and this was confirmed by Alizarin red staining over a 34-day time-course (Fig. 1C and D, Fig. 3A and B). Quantification of this staining indicated that after an initial delay, presumably while early differentiation stages were occurring, calcium accumulation increased rapidly from Day 17 to 34 (Fig. 3B) (P<0.001).

Levamisole, a well established inhibitor of ALP, inhibited ATDC5 ECM mineralisation at Day 15 of culture at concentrations in excess of 300 μM (P<0.001) (Fig. 3C) with no apparent alterations in the morphology of the ATDC5 cells (Fig. 3D) (13). This indicated that the enzyme ALP is required, and therefore that chondrogenic differentiation of the ATDC5 cells is necessary for effective mineralisation.

**Figure 4.**
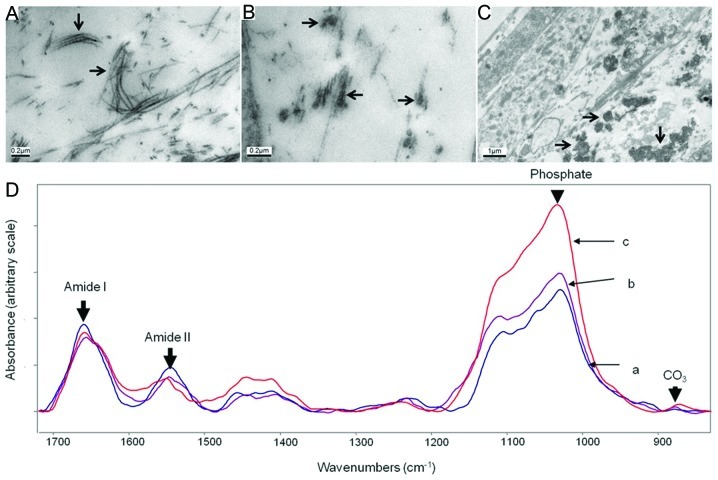
Analysis of ATDC5 mineral deposition. TEM images of ATDC5 cultures at Day 16 of culture. (A) Collagen fibers are present in the ECM; arrows denote collagen fibers. (B) Mineral deposition is noted along collagen fibers; arrows indicate an electron-dense material, likely to be mineralisation spreading along the collagen fibers within the ECM. (C) Mineral deposition within the ECM; arrows indicate electron-dense mineralised regions of the ECM. (D) FTIR analysis of (a) ATDC5 monolayer at 41 days of culture; (b) mineralised embryonic mouse bone at E14; (c) cortical bone from the tibia of a 4-month-old mouse. Absorbances of the phosphate peaks (900–1200 cm^−1^), which represents mineralisation and the amide-I peak (1585–1725 cm^−1^), which indicates protein, were used to estimate the mineral-to-matrix ratio of the mineralised ECM. Absorbance values for each plot are arbitrary and not to scale.

FTIR and TEM were adopted as two well recognised methods to determine whether the properties of the mineral formed in culture is similar to that which is formed by mineralised cartilage *in vivo* (14). ATDC5 cells were shown to produce a collagenous ECM by TEM in which banded fibers, synonymous with collagen fibers, were present in the ECM (Fig. 4A) (15). Along some of the collagen fibers, electron-dense regions were present, indicative of the onset of mineralisation (Fig. 4B). In some discrete regions of the ECM, electron-dense spheres of ∼200–500 nm were present which were also associated with the collagenous fibers (Fig. 4C); these are possibly mineralised matrix vesicles (MVs) (1,8,9). These results suggest that ATDC5 cells produce a collagenous ECM and that the mineral formed is in alignment with the collagen fibrils, as is observed in endochondral ossification. ATDC5 cells were cultured for 41 days for FTIR analysis (Fig. 4D-a). The FTIR spectra were compared with those of E14 embryonic mouse bone (Fig. 4D-b) and 4-month-old cortical mouse bone (Fig. 4D-c), and were used to generate numerical parameters which may be compared with *in vivo* samples (Table I). The resulting data strongly suggest that the mineralisation of the ATDC5 monolayers resembles that of embryonic mouse bone.

## Discussion

Attempts to unravel the underlying mechanisms of endochondral ossification have been limited by current models. The data presented in this manuscript characterise a novel, rapid culture method for studying physiological chondrocyte ECM mineralisation using ATDC5 cells that we observed to be highly reproducible. A mineralisation method for ATDC5 cell culture was first described by Shukunami *et al* (3), however its drawbacks have been identified by several other groups and thus the method has been gradually developed with time. In this study, we cultured ATDC5 cells in the presence of ascorbic acid and 10 mM βGP.

ATDC5 cells have previously been cultured with ascorbic acid, which facilitates collagen synthesis. This reduces the proliferation phase of the cells and promotes their differentiation (6,16). Ascorbic acid has also been shown to promote the hypertrophic differentiation of cultured primary chick chondrocytes (17). In the present study, cells were cultured in the presence of 50 μg/ml ascorbic acid from when they reached confluency and in concurrence with previous studies, this promoted ECM formation. The increased mRNA expression of the chondrogenic marker *Col2a1* correlated with the onset of Alcian blue-stained cartilaginous nodules and the increased mRNA expression of *Col10a1* with the differentiation of the cells to a hypertrophic phenotype. The delayed onset of *Col10a1* transcription at Day 10 is consistent with the 2 stages of differentiation that must occur from Day 6 for the cells to become hypertrophic. The observation that *Col10a1* expression preceded the first observations of mineral formation provides further evidence that the model is able to mimic the *in vivo* endochondral ossification. During differentiation the histology of the cultures was similar to that described by Shukunami *et al* (3).

In addition to ascorbic acid, an exogenous phosphate source is routinely added to cell cultures to induce and stimulate mineralisation of the ECM. βGP is a preferential exogenous organic phosphate source as it is a substrate for ALP and therefore the cells directly dictate when it is cleaved to release inorganic phosphate with their differentiation to a hypertrophic phenotype. In this study we cultured ATDC5 cells in the presence of 10 mM βGP and observed mineral formation from Day 15 of culture upon collagen fibrils and within MVs. However, in a number of osteoblast and chondrocyte cultures, the growth of cells in the presence of βGP has been shown to lead to the formation of sporadic mineral formation on the cell surface and in the culture medium and not upon collagen fibrils which is regarded to be dystrophic mineralisation and not physiological hydroxyapatite (18,19).

In the present study we showed that the presence of ALP is necessary for βGP-induced ATDC5 mineralisation. This is consistent with previous studies in which the activity of ALP has been investigated in ATDC5 cells (3). Furthermore, the addition of levamisole, a potent inhibitor of ALP, to ATDC5 cultures inhibited their ECM mineralisation. Mineralisation was also inhibited in cells cultured in the presence of βGP and in the absence of insulin, which is required for their differentiation (data not shown) (20). This result, therefore, suggests that mineral formation is dependent upon both chondrogenic differentiation and the subsequent presence of ALP. Additionally, the inhibition of ECM mineralisation when ATDC5 cells were cultured without insulin further emphasises that the mineral formed is not dystrophic and is dependent on their differentiation status.

Although routinely used as indicators of mineralisation, Alizarin red and von Kossa staining are not sufficient to conclude that mineralisation is physiological since the presence of calcium and/or phosphate does not indicate HA formation *per se* (21). For this reason, we adopted FTIR and TEM to examine whether or not the mineral formed in culture is physiological (14).

Shukunami *et al* (3) have previously used TEM for ATDC5 analysis and reported the presence of extremely dark calcium-containing spherites which they identified as mineralised MVs in discrete regions between the cells, associated with collagenous fibers. In the present study, structures were noted in our TEM analysis which were indistinguishable in shape and size from those reported by Shukunami *et al* (3). The MVs derived from ATDC5 cells are of a similar size and appearance to MVs derived from *in vivo* tissues including chicken growth plate chondrocytes and rat epiphyseal hypertrophic chondrocytes (22). These results indicate that the ultrastructure of the collagenous fibrils appears as expected and that mineralisation of ATDC5 cultures appears to form in a physiological manner.

Furthermore, in this study we showed that the spectra of the ATDC5 monolayer more closely resembled that of the developing embryonic bone compared to the fully developed cortical bone. The mineral-to-matrix ratio of the ATDC5 cultures, a key determinant of mineral composition is similar to the values for mineralised embryonic bone, E14, which is the earliest point at which mineralisation occurs in the mouse (23). The mineral-matrix ratios in other publications provide additional comparisons: in 10-day-old and 10-week-old wild-type mouse calcified growth plate cartilage the ratios have been calculated as 2.7 and 5.48, respectively (24,25). There is a considerable variation in these parameters, but the mineral-matrix ratio within the ATDC5 monolayer is within the expected region. The ATDC5 monolayer model characterised by Shukunami *et al* (3) was analysed by FTIR and spectra were compared with those of cultured primary rabbit chondrocytes, resulting in spectra which were almost super-imposable. These spectra show that the absorbance is greater in the amide-range compared to the phosphate-range, thus although the mineral-to matrix ratio is not provided, it is certainly less than those reported in the present study. Therefore, ECM mineralisation is greater in this ATDC5 model compared to the method generated by Shukunami *et al* (3). If mineralisation in the cultures is ectopic and mineral is accumulated simply due to ALP cleavage of βGP, the ratio of mineral-to-matrix would be expected to be extremely high, which is not the case. A study by Huitema *et al* (26), demonstrated this; inorganic phosphate was added to medium conditioned by ATDC5 cells which generated flat, mineralised structures, with extremely small amide-I peaks, relative to phosphate peaks.

The mineralised product of the ATDC5 monolayer produced a phosphate peak with a clear shoulder at approximately 1,130 cm^−1^. This is characteristic of the hydroxyapatite containing acid phosphate which is gradually lost as the crystal matures; thus both the ATDC5 monolayer and the embryonic bone contain this peak, which is absent in the mature hydroxyapatite sample (27,28). The values obtained from the carbonate substitution and crystallinity are within the range of biologically relevant *in vivo* samples, which also indicates that this ATDC5 model generates physiologically relevant mineral (24,29).

The development and characterisation of a rapidly mineralising chondrocyte model has the potential to assist us in better understanding the underpinning molecular mechanisms responsible for poor linear bone growth which is observed in a number of chronic diseases such as cystic fibrosis, chronic kidney disease, rheumatological conditions and inflammatory bowel disease. Chondrocyte models, including the ATDC5 cell line, have proved invaluable for determining the effects of pro-inflammatory cytokines and glucocorticoids on chondrocyte proliferation, differentiation and gene expression (9,10). However, the absence of a practical and accessible *in vitro* chondrocyte mineralisation model has hindered a fuller appreciation of how cartilage mineralisation and endochondral ossification are disrupted by factors e.g. cytokines and drugs, that are responsible for impaired linear bone growth in children.

In conclusion, in this study we developed and characterised an improved and rapid method of ATDC5 differentiation which develops a physiologic mineralised ECM 15 days after seeding. To our knowledge, this is the earliest report of mineralisation in which physiological attributes of the mineral have been characterised.

## Figures and Tables

**Table I.** Mineralisation parameters from FTIR samples.

Sample	Mineral-to-matrix ratio	Carbonate-to-mineral ratio	Crystallinity
A	3.000±0.917	0.008±0.005	1.128±0.009
B	2.200±2.500	0.005±0.002	1.072±0.062
C	6.500±0.900	0.006±0.001	1.130±0.030

Using peak areas from FTIR spectra, the mineral-to-matrix ratio (900–1,200 cm^−1^/1,585–1,725 cm^−1^), carbonate-to-mineral ratio (850–950 cm^−1^/900–1,200 cm^−1^) and crystallinity (1,030 cm^−1^/1,020 cm^−1^) were calculated for mineralised regions of (A) ATDC5 monolayer grown to 41 days of culture, (B) mineralised E14 mouse bone and (C) ortical bone from the tibia of a 4-month-old mouse.
